# HIV drug resistance and treatment success in the Republic of Congo: Implications for optimized treatment

**DOI:** 10.1111/hiv.70261

**Published:** 2026-05-29

**Authors:** Dominic Rauschning, Arcy Marcelin Elenga Ike, Johanna Holterhoff, Amira Stockinger, Darrel Ornelle Elion Assiana, Claujens Chastel Mfoutou Mapanguy, Freisnel Hermeland Mouzinga, Carola Horn, Isabelle Suarez, Ferdinand Heyn, Diane Alexia Fila, Hubert Banguissa, Fabien Roch Niama, Donatien Moukassa, Eva Heger, Francine Ntoumi, Clara Lehmann

**Affiliations:** ^1^ Department I of Internal Medicine University Hospital of Cologne Cologne Germany; ^2^ Department IB of Internal Medicine Bundeswehr Central Hospital Koblenz Germany; ^3^ Fondation Congolaise pour la Recherche Médicale Brazzaville Republic of Congo; ^4^ Faculté des Sciences de la Santé de l’Université Marien Ngouabi Brazzaville Republic of Congo; ^5^ Département de Biologie Médicale Hôpital central des Armées Pierre Mobengo Brazzaville Republic of Congo; ^6^ Institute of Virology, University Hospital of Cologne Cologne Germany; ^7^ Laboratoire National de Référence des Mycobactéries Brazzaville Republic of Congo; ^8^ Programme National de Lutte contre la Tuberculose Brazzaville Republic of Congo; ^9^ Boulevard Denis Sassou Nguesso Brazzaville Republic of Congo; ^10^ German Center for Infection Research (DZIF), partner site Bonn‐Cologne Cologne Germany; ^11^ Association Avenir Positif Pointe‐Noire Republic of Congo; ^12^ Laboratoire National de Santé Publique Brazzaville Republic of Congo; ^13^ Institute of Tropical Medicine, University of Tübingen Tübingen Germany

**Keywords:** ART, drug resistance, HIV, Republic of Congo, sub‐Sahara Africa

## Abstract

**Objectives:**

Increasing rates of HIV‐1 drug resistance (HIVDR) threaten the effectiveness of antiretroviral therapy (ART) programmes in sub‐Saharan Africa, particularly among children, adolescents and young adults, who face limited treatment options. The Republic of Congo (ROC) lacks comprehensive national data on HIVDR in these vulnerable populations. The study aimed to determine the proportion of virologically unsuppressed young individuals receiving ART in ROC and to characterize the prevalence and patterns of HIVDR.

**Methods:**

A point prevalence analysis was conducted in Pointe‐Noire to determine viral load and to characterize resistance‐associated mutations (RAMs) in HIV‐1 among children, adolescents and young adults receiving ART using next‐generation sequencing. Sociodemographic and clinical data were collected to assess associations between resistance, treatment history and adherence.

Ethical approval was obtained (Avis n° 034/CIE/FCRM/2021).

**Results:**

A total of 510 participants were included, with a median age of 19.5 (IQR 13–40) and a median CD4 T‐cell count of 538/μl (IQR 279–901). Among them, 34% (*n* = 173) had detectable viral loads (> 40 copies/ml) while on ART. Of the viremic cohort, 147 samples were successfully sequenced and showed diverse HIV subtypes, with subtype A1 (21%) being the most prevalent. Drug resistance was most frequently observed against NRTIs and NNRTIs, with M184V (54.1%) and K103N (40.8%) mutations predominating. Resistance to protease inhibitors was rare. Notably, no major resistance was found to integrase inhibitors or capsid inhibitors.

**Conclusions:**

HIV‐1 drug resistance is highly prevalent in young people receiving ART in the ROC. This study highlights the urgent need to integrate HIVDR genotyping into clinical practice in the ROC to guide treatment and preserve future treatment options. Strengthened adherence support, resistance testing at treatment failure and equitable access to newer antiretroviral drugs are essential to sustain treatment effectiveness and support progress toward the 2030 global HIV targets.

## INTRODUCTION

HIV drug resistance (HIVDR) poses an escalating threat to the success of antiretroviral therapy (ART) programmes in sub‐Saharan Africa. Recent data reveal that pretreatment resistance to non‐nucleoside reverse transcriptase inhibitors (NNRTIs) approaches or exceeds 10% across much of the region, with particularly pronounced increases in southern and eastern Africa [1, 2]. The World Health Organization (WHO) warns that the durability of current first‐line regimens depends on early detection and continuous surveillance of resistance mutations [1].

In settings where programmatic transitions to dolutegravir (DTG)‐based regimens are ongoing, monitoring the evolving landscape of resistance is essential to preserve treatment efficacy and inform national HIV management strategies.

In the Republic of Congo (ROC), the estimated HIV prevalence among adults is 3.2%, with ART coverage exceeding 70% [3]. Despite significant public health achievements, virological failure and regimen changes remain frequent, with rates above 30% in ROC [4, 5]. While earlier data from 2004 suggested a resistance prevalence of 4% [6], more recent reports from neighbouring Central African countries indicate increasing resistance‐associated mutations (RAMs) to (N)NRTIs, reflecting prolonged exposure to legacy first‐line therapies [7, 8]. Currently, first‐line ART in ROC mainly consists of DTG in combination with two NRTIs according to WHO guidelines [9]. With the ongoing transition to DTG‐based ART in ROC, monitoring resistance patterns and evaluating treatment outcomes is therefore essential for optimizing this programmatic shift.

Central Africa harbours the greatest HIV‐1 genetic diversity globally, with all major subtypes, numerous circulating and unique recombinant forms, which may influence viral fitness, drug susceptibility and diagnostic assay performance [6, 10, 11, 12, 13, 14]. Moreover, sociobehavioral determinants such as treatment interruptions, stigma and limited psychosocial support contribute to virological failure and the selection of resistant variants [15]. Understanding the interplay between virological, genotypic and behavioural factors is therefore critical for optimizing ART outcomes.

This study aimed to determine a treatment success rate and characterize the prevalence and patterns of HIVDR, focusing on children, adolescents and young adults receiving ART in ROC. Using next‐generation sequencing (NGS) and comprehensive socioclinical data, we assessed RAMs across multiple viral gene regions and evaluated their relationship with treatment history, adherence, ART regimens and sociogeographic factors. These findings provide a detailed updated molecular resistance profile from ROC and offer evidence‐based guidance for national ART policy and future implementation of long‐acting therapies.

## MATERIALS AND METHODS

### Study population and data acquisition

**FIGURE 1:**
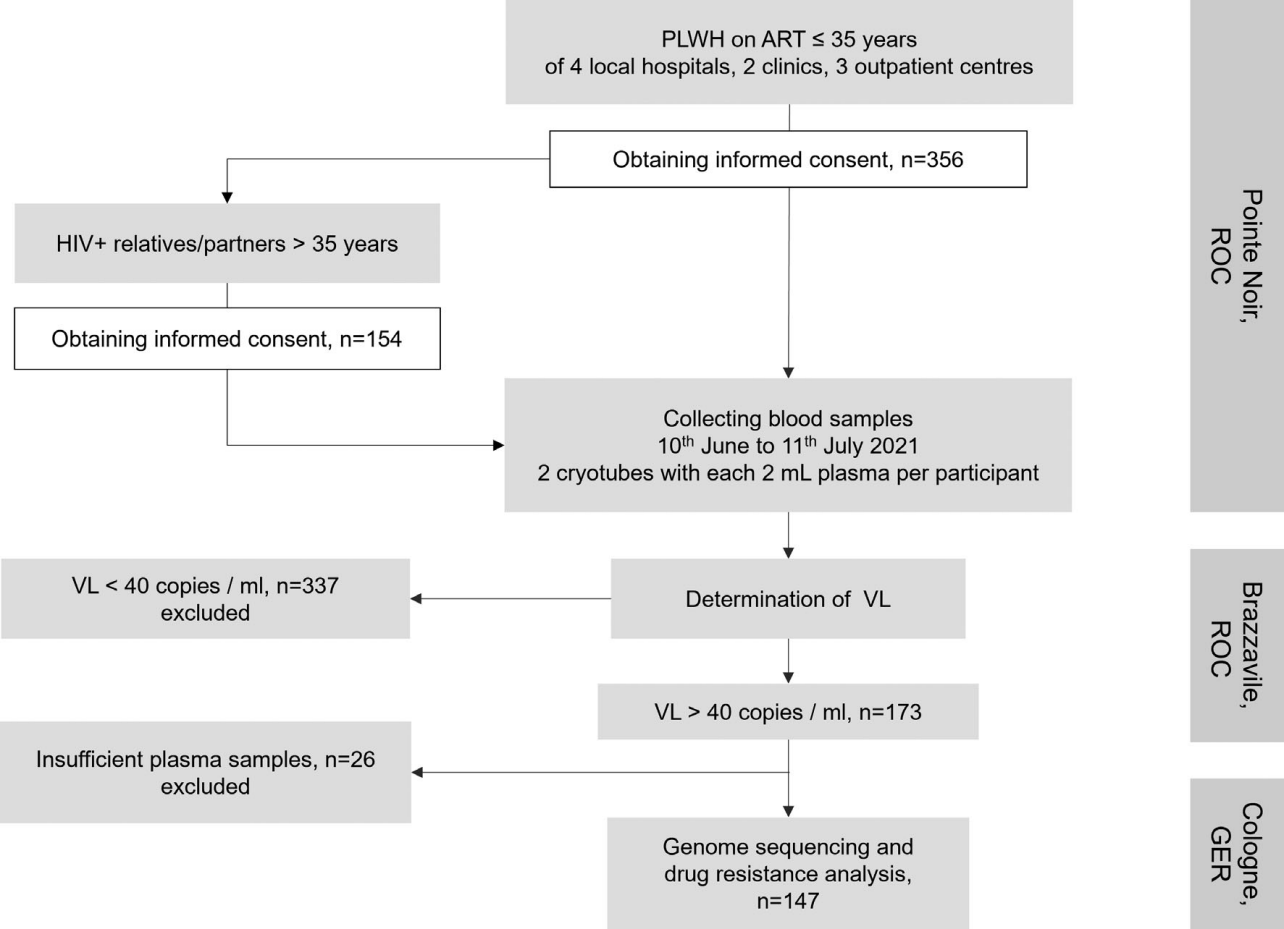
Outline of the study design. ART, antiretroviral therapy; GER, Germany; PLWH, people living with HIV; ROC, Republic of Congo; VL, viral load.

**FIGURE 2:**
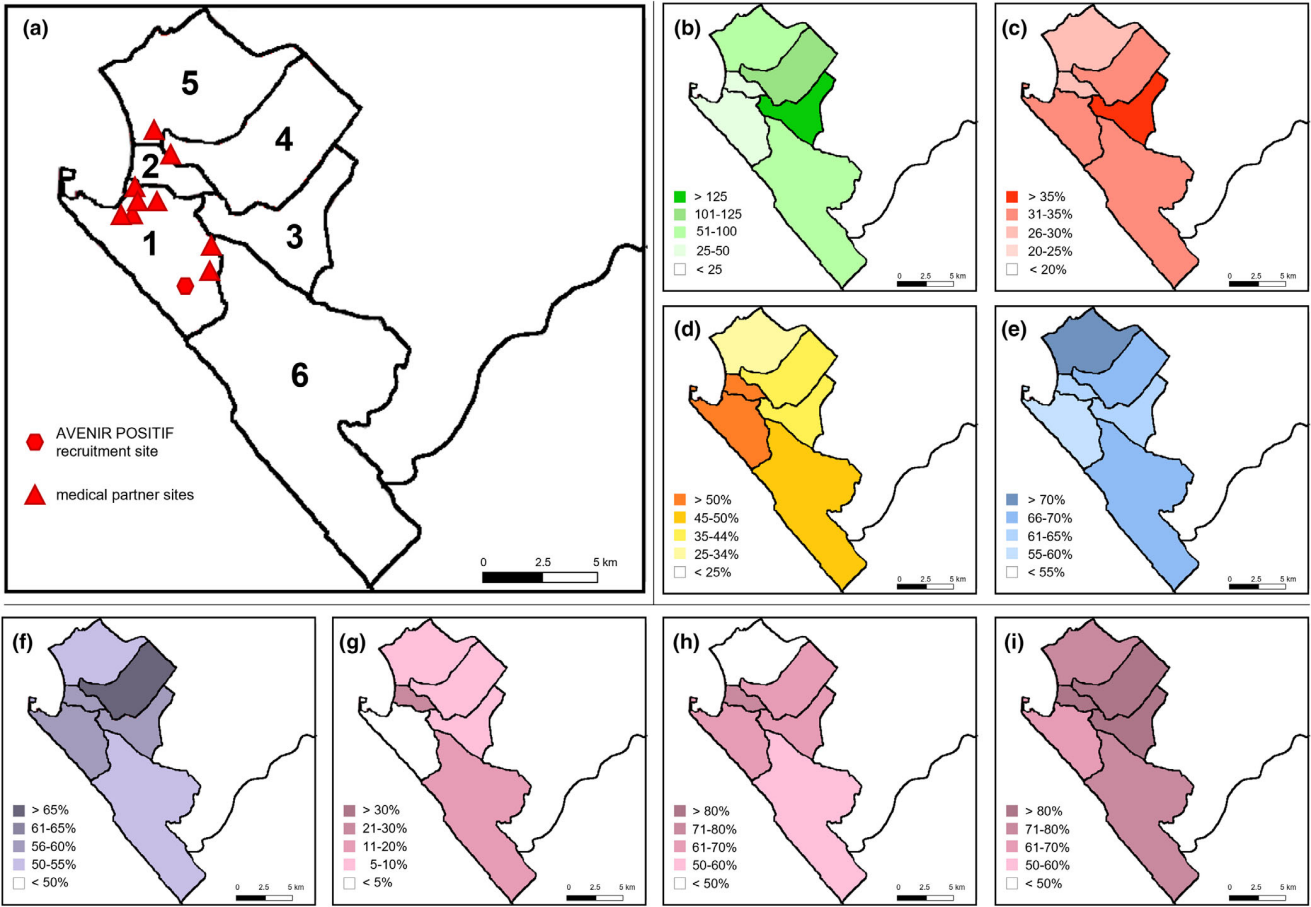
Location of the recruitment site and participating hospitals and medical centres in the city of Pointe‐Noire with its six arrondissements (a). Distribution of the participants (absolute number) (b); distribution of participants with detectable VL (proportion of participants living in the arrondissement) (c); distribution of participants with INSTI‐based ART regimen (proportion of participants living in the arrondissement) (d); distribution of participants obtaining psycho‐social support (proportion of participants living in the arrondissement) (e); distribution of participants with resistance to at least one ARV‐class (proportion of participants with detectable VL living in the arrondissement) (f); distribution of participants with resistance to PIs (proportion of participants living in the arrondissement*) (g); distribution of participants with resistance to NRTIs (proportion* of participants living in the arrondissement*) (h); distribution of participants with resistance to NNRTIs (proportion of participants living in the arrondissement*) (i). *Proportions in subfigures G–I are related to participants with detectable VL and successfully amplified HIV genome region living in the arrondissement. Exact numbers graphically interpreted in subfigures B–I can be found in Supplements S9 and S10. ART, antiretroviral therapy; ARV, antiretroviral; INSTI, integrase inhibitor; NNRTI, non‐nucleoside reverse transcriptase inhibitor; NRTI, nucleoside reverse transcriptase inhibitor; PI, protease inhibitor; VL, viral load.

The study was a cross‐sectional study of young individuals living with HIV‐1 infection on ART at Pointe‐Noire, ROC. All study participants were recruited by the charitable, HIV care‐providing non‐governmental organization AVENIR POSITIF in Pointe‐Noire between 10 June 2021 and 11 mate July 2021 (Figures 1 and 2a).

Data were collected using venous blood samples and standardized questionnaires. Furthermore, patient files of AVENIR POSITIF were analysed concerning the anamnestic antiretroviral treatment. The study primarily included all individuals up to the age of 35 who were receiving ART and submitted a completed questionnaire. In order to analyse possible familial transmission routes, older relatives and partners living with HIV were also included when they were also receiving ART and submitted a completed questionnaire (Figure 1).

### Laboratory methods

Two 5 mL EDTA tubes of venous blood were taken from each study participant and processed on site, obtaining two tubes of plasma aliquots of 2 mL each (Figure 1). After being stored at −20°C during the recruitment phase at Pointe‐Noire, all samples were transported to Brazzaville, ROC, for further processing while maintaining the cold chain.

The viral load (VL) testing was performed at the Fondation Congolaise pour la recherche médicale (FCRM) laboratory in Brazzaville. The VL of the first 61 samples was determined using the cartridge‐based GeneXpert polymerase chain reaction (PCR) system (Cepheid, Sunnyvale, California, USA). Due to the temporary unavailability of GeneXpert cartridges, the remaining 449 samples were analysed at the National Public Health Laboratory of the Republic of Congo, in Brazzaville, using the Abbott m2000sp Realtime System PCR and Sample Preparation (Abbott, Abbott Park, Illinois, USA). Although combining VL platforms may introduce some degree of measurement variability, this approach was unavoidable under real‐world operational conditions. Both systems are WHO‐prequalified, widely used and have shown comparable performance for VL monitoring [16, 17]. All analyses were conducted according to the manufacturer's instructions.

Samples with detectable VL (> 40 copies/mL) and a remaining plasma residue of more than 1 mL were transferred for resistance testing to the University Hospital Cologne, Germany, in compliance with international transport regulations (Figure 1).

After amplification of HIV RNA by conventional PCR at the Institute of Virology at Cologne University Hospital, following the in‐house protocol with a self‐designed primer panel, NGS sequence analysis was outsourced to Seq It GmbH & Co.KG, Kaiserslautern, Germany, using the Illumina system (Illumina Inc., San Diego, California, USA). As per local routine protocol, sequence data were analysed to determine the HIV subtype, tropism and RAMs as well as the resistance level against typical antiretrovirals (ARVs) at a 10% threshold [18] using the Stanford University HIV Drug Resistance Database [19], HIV‐GRADE (https://www.hiv-grade.de/), geno2pheno (https://www.geno2pheno.org/) and COMET [20].

### Statistical analysis

In addition to comparing individual characteristics, particularly the ART regimens used, the documented resistances were statistically analysed. A subgroup analysis was performed on participants with family connections to each other, as well as a locoregional analysis of the city of Pointe‐Noire. Characteristics are shown as absolute numbers and percentages, medians plus interquartile range (IQR) or means plus/minus standard deviation (SD), as appropriate. Categorical variables are presented in frequencies and percentages; comparison was performed by *χ* ^2^‐test for independence or Fisher's exact test. Continuous variables were analysed using Mann–Whitney‐*U*‐Test or *t*‐test as appropriate. *p*‐Values <0.05 were considered statistically significant, demanding 95% confidence intervals (95% CI). IBM SPSS Statistics (Version 29.0.2.0, IBM Corporation, New York, USA) was used for all analyses.

## RESULTS

The study included a total of 510 participants with a median age of 19.5 years (IQR 13–40). The majority of participants were women (311/510, 61%). The median body mass index (BMI), as a surrogate for physical constitution, was 19.5 (IQR 16–23). The participants had acquired HIV a median of 10 years ago (IQR 5.3–13.5) and had been receiving ART for 9 years (IQR 4.4–13.3). According to the WHO classification, the majority were in stage I of HIV infection (448/510, 87.8%). The median last ever before enrollment documented CD4 cell count was 538/μl (IQR 279–901), and the participants had already taken at least one ART regimen in the past on average. Table 1 and Supplements S1 and S2 provide an overview of the demographic and clinical characteristics of the cohort.

**TABLE 1:** Demographic and clinical characteristics of the participants.

Characteristics	Participating PLWH on ART			
	Detectable	Not detected	*p*‐Value	Total
	Viral load	Viral load		
	*n* = 173	*n* = 337		*n* = 510
Sex				
Male	65 (37.6)	134 (39.8)	0.631	199 (39.0)
Female	108 (62.4)	203 (60.2)		311 (61.0)
Median age, years [IQR]	18.0 [13–33]	20.0 [14–42]	**0.036**	19.5 [13–40]
Median body mass index, kg/m^2^ [IQR]	18.4 [15–22]	20.0 [17–23]	**0.006**	19.9 [16–23]
Employment status			0.181	
Employed	45 (26.0)	108 (32.1)		153 (30.0)
Unemployed/other^a^	123 (71.1)	223 (66.2)		346 (67.8)
Missing	5 (2.9)	6 (1.8)		11 (2.2)
Educational level				
None/primary school	58 (33.5)	105 (31.2)	0.587	163 (32.0)
Secondary school/higher	115 (66.5)	232 (68.8)		347 (68.0)
Marital state				
No partner/widow	143 (82.7)	259 (76.9)	0.129	402 (78.8)
In partnership	30 (17.3)	78 (23.1)		108 (21.2)
Median duration of HIV infection, years [IQR]	9.5 [5.4–13.4]	10.4 [5.1–13.8]	0.823	10.4 [5.3–13.5]
Median time since ART start, years [IQR]	8.5 [4.5–12.8]	9.5 [4.4–13.4]	0.675	9.4 [4.4–13.3]
HIV type			0.233	
HIV‐1	97 (56.1)	190 (56.4)		287 (56.3)
HIV‐2	2 (1.2)	1 (0.3)		3 (0.6)
Missing	74 (42.8)	146 (43.3)		220 (43.1)
Viral load at recruitment				
Median, cop/ml [IQR]	2582 [369–17 821]	‐		‐
40–200 cop/ml	23 (13.3)	‐		‐
201–1000 cop/ml	41 (23.7)	‐		‐
>1000 cop/ml	109 (63.0)	‐		‐
Median last CD4 cell count, /μl [IQR] (missing *n* = 369)	478 [283–893]	622 [278–917]	0.550	538 [279–901]
<12 months before recruitment (*n* = 7)	1400 [5–1400]	165 [39–804]		212 [13–1400]
≥12 months before recruitment (*n* = 134)	477 [289–867]	623 [310–920]		538 [303–880]
WHO HIV stage			0.072	
Stage 1	144 (83.2)	304 (90.2)		448 (87.8)
Stage 2	15 (8.7)	18 (5.3)		33 (6.5)
Stage 3	14 (8.1)	15 (4.5)		29 (5.7)
HIV mode of transmission			**0.015**	
Vertical	95 (54.9)	162 (48.1)		257 (50.4)
Sexual	46 (26.6)	132 (39.2)		178 (34.9)
Other/unknown	32 (18.5)	43 (12.8)		75 (14.7)
Reason for initial HIV test			0.377	
Self‐initiated/voluntary	36 (20.8)	85 (25.2)		121 (23.7)
Other	62 (35.8)	117 (34.7)		179 (35.1)
Unknown	75 (43.4)	135 (40.1)		210 (41.2)
Maternal HIV status			**0.016**	
Negative	20 (11.6)	68 (20.2)		88 (17.3)
Positive	85 (49.1)	145 (43.0)		230 (45.1)
Unknown	68 (39.3)	124 (36.8)		192 (37.6)
Paternal HIV status			0.808	
Negative	40 (23.1)	104 (30.9)		144 (28.2)
Positive	22 (12.7)	53 (15.7)		75 (14.7)
Unknown	111 (64.2)	180 (53.4)		291 (57.1)
HIV/TB co‐infection	31 (17.9)	66 (19.6)	0.650	97 (19.0)
HIV/HBV co‐infection	1 (0.6)	‐	‐	1 (0.2)
HIV/HCV co‐infection	‐	1 (0.3)	‐	1 (0.2)
Interruption of ART in the past	115 (66.5)	186 (55.2)	**0.014**	301 (59.0)
Median number of ART regimens [IQR]	2 [2–2]	2 [2–2]	0.490	2 [2–2]
Median number of tablets daily taken, [IQR]	1 [1–4]	1 [1–3]	**0.016**	1 [1–3]
Patient of AVENIR POSITIF				
Yes	127 (73.4)	211 (62.6)	0.301	338 (66.3)
No	2 (1.2)	1 (0.3)	**(0.015** ^b^ **)**	3 (0.6)
Missing	44 (25.4)	125 (37.1)		169 (33.1)
Obtaining psycho‐social support				
Yes	127 (73.4)	212 (62.2)	**0.019**	339 (66.5)
Therapy education	123 (71.1)	200 (59.3)	**0.009**	323 (63.3)

*Note*: Categorical variables, *n* (%), were compared by *χ* ^2^‐test for independence. Comparison of medians used Mann–Whitney‐*U*‐test. Statistical significance is considered at *p*‐values <0.05 (Bold values indicate statistically significant values), demanding 95% confidence intervals.

Abbreviations: ART, antiretroviral therapy; ENV, HIV envelope gene; HBV, hepatitis B virus; HCV, hepatitis C virus; IQR, interquartile range; PLWH, people living with HIV; TB, tuberculosis; WHO, World Health Organization.

^a^ Including students, housewives and retired.

^b^ *p*‐Value, if missing cases were classified as “no”.

Overall, 173 (33.9%) individuals had a VL above the detection limit, and 337 (66.1%) participants were virologically suppressed (Table TABLE 1). Age and body weight differed significantly between the two groups, with unsuppressed participants having a lower median age (18.0 years, IQR 13–33 vs. 20.0 years, IQR 14–42; *p* = 0.036) and a lower median BMI (18.4 kg/m^2^, IQR 15–22 vs. 20.0 kg/m^2^, IQR 17–23; *p* = 0.006) (Table TABLE 1).

The group with detectable VL had more often interrupted their ART (11/173, 67.6% vs. 186/337, 55.2%; *p* = 0.014) and showed a significantly lower rate of sexually acquired HIV infections (46/173, 26.6% vs. 132/337, 39.2%; *p* = 0.015). Maternal HIV status differed between the groups overall (*p* = 0.016), with a lower proportion of participants reporting a negative maternal HIV status in this group (20/173, 11.6% vs. 68/337, 20.2%) (Table TABLE 1).

With regard to psycho‐social support, obtaining support differed significantly between PLWH with detectable and suppressed VL (127/173, 73.4% vs. 212/337, 62.2%; *p* = 0.019). In particular, therapy education was significantly more often received among PLWH with detectable VL (123/173, 71.1% vs. 200/337, 59.3%; *p* = 0.009). Interestingly, significantly more members of this group did not provide any information about their relation to AVENIR POSITIF (44/173, 25.4% vs. 125/337, 37.1%; *p* = 0.030). As this charitable association gave care to approximately 340 people at the time of the study, the missing data can be interpreted as a ‘no’.

### ART

**FIGURE 3:**
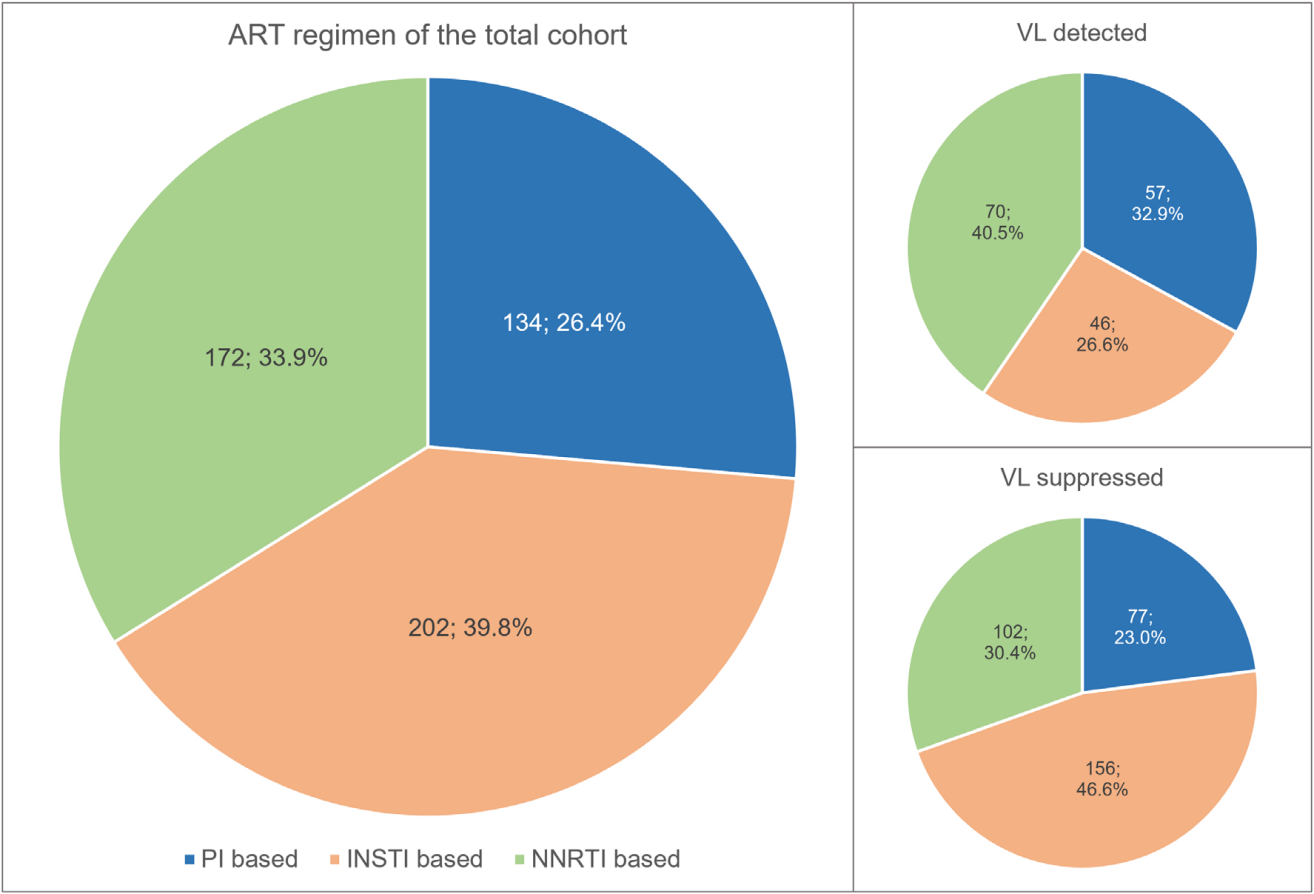
Distribution of ART regimens among the cohort and divided by PLWH with (*n* = 337, 2 missings) or without (*n* = 173) viral suppression. *n*; %. INSTI, integrase inhibitor; NNRTI, non‐nucleoside reverse transcriptase inhibitor; PI, protease inhibitor; VL, viral load.

Looking at the ART regimens used in the cohort, the integrase inhibitor (INSTI) based regimen consisting of tenofovir disoproxil fumarate (TDF) + lamivudine(3TC) + dolutegravir (DTG) was the most commonly used at 38.6% (197/510), followed by the NNRTI based regimen of TDF + FTC + EFV (96/510, 18.8%) and the protease inhibitor (PI) based regimen consisting of abacavir (ABC) + 3TC + ritonavir (r)‐boosted lopinavir (LPV) (79/510, 15.5%) (Supplement S3). Figure 3 shows the distribution of ART regimens classified according to the relevant drug classes PI, INSTI and NNRTI in the overall cohort, but also in the subgroups of virologically suppressed participants and participants with detectable VL. The statistical analysis revealed significant differences in the subgroups: PLWH with detectable VL were more frequently on boosted PI and NNRTI compared to the group with suppressed VL (*p* = 0.016 and *p* = 0.024, respectively) (Supplement S4). In contrast, PLWH with suppressed VL were significantly more often on INSTI‐based ART (*p* < 0.001) (Supplement S4). With regard to individual active substances, it was found that LPV/r was significantly more likely to be part of an insufficient regimen (*p* = 0.011), as was FTC (*p* = 0.005) (Supplement S4). On the other hand, DTG was significantly more likely to be associated with successful ART (*p* < 0.001) (Supplement S4).

### Resistance to ART

For the resistance analysis, 147 blood samples with detectable VL (147/173, 85.0%) could be used (Figure 1). Subtype A1 was the most prevalent (31/147; 21.1%), followed by subtype H (18/147; 12.2%) and G (17/147; 11.6%). A total of 9 different subtypes could be clearly identified (Supplement S5).

The protease reverse transcriptase (PrRT) region was amplified in 98 (98/147, 66.7%), integrase (INT) region in 101 (101/147, 68.7%), group specific antigen (GAG) region in 116 (116/147, 78.9%) and envelope (ENV) region in 100 samples (100/147, 68.0%). Overall, 85 (57.8%) of 147 viremic participants showed resistance to at least one of the ARVs tested, including capsid inhibitors (CAIs) and entry inhibitors (EIs). In this subgroup, HIV‐subtype H and viruses with R5‐tropism were more likely observed than in sequenced samples without HIVDR, hence HIV subtype and tropism showed no overall statistically significant association with the detection of HIVDR (*p* = 0.470 and *p* = 0.053; Supplement S5). Moreover, the sequenced samples with HIVDR showed a higher median VL (6164 cop/ml vs. 588 cop/ml; *p* < 0.001; Supplement S5). Supplement S6 presents the detected resistance‐associated mutations to ARV classes.

Only a few major mutations associated with PI resistance were observed, and only accessory mutations in INSTIs and CAIs were found. In contrast, a large number of different mutations mediating (N)NRTI resistance were observed. The most common mutation associated with NRTI resistance was M184V (53/98, 54.1%). K103N was the most frequently detected resistance mutation for NNRTIs (40/98, 40.8%) (Supplement S6). Some RAMs were detected against the EI fostemsavir (FTR), although this substance is not used in ROC (Supplement S6).

**FIGURE 4:**
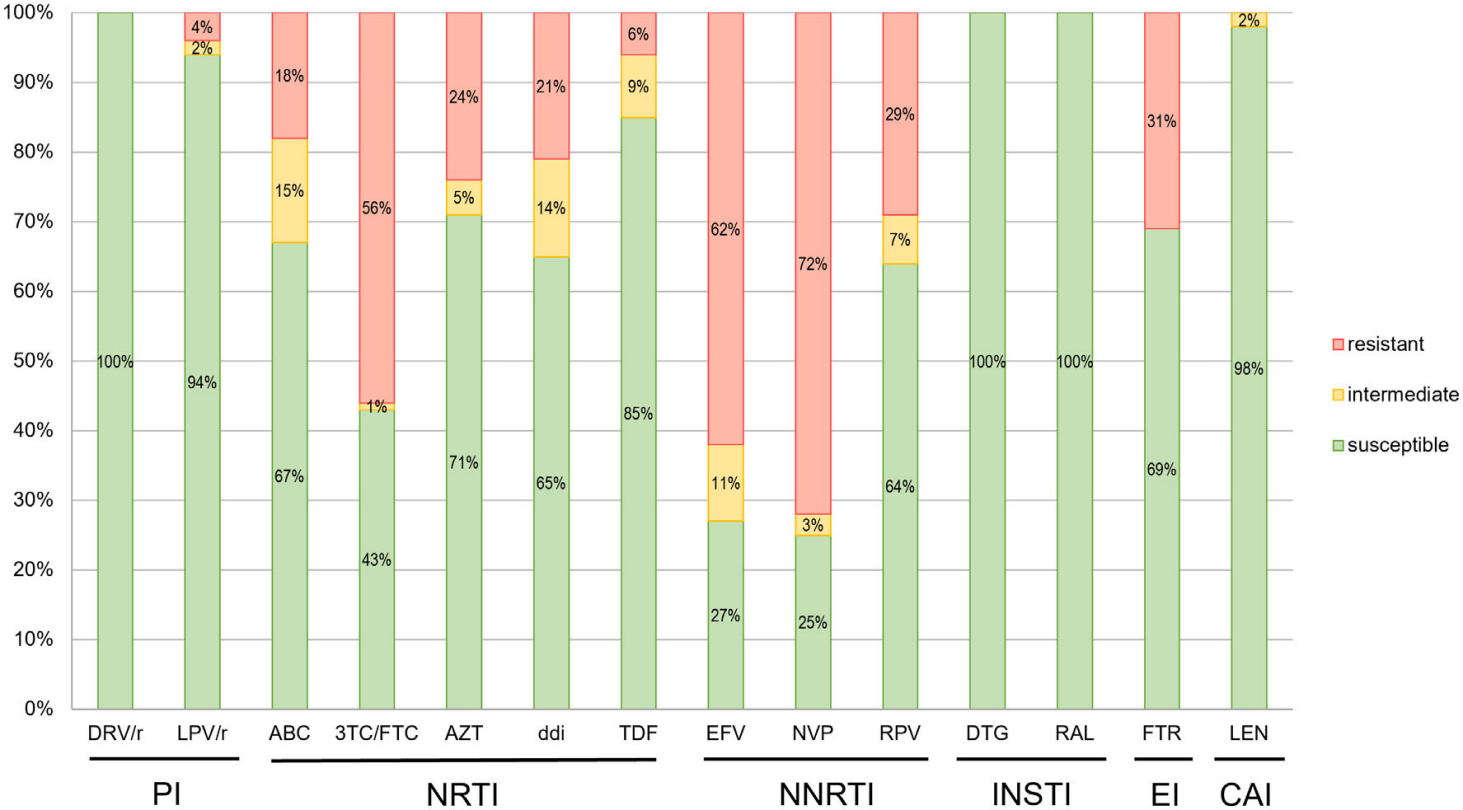
Distribution of **r**esistance levels of ARVs of different substance classes. Percentages refer to the number of samples with the corresponding, successfully amplified genome region where resistance‐associated mutations to the substance class are encoded. ABC, Abacavir; ARV, antiretroviral; AZT, Zidovudine; CAI, capsid inhibitor; ddi, Didanosin; DRV, Darunavir; DTG, Dolutegravir; EI, entry inhibitor; EFV, Efavirenz; FTC, Emtricitabine; FTR, Fostemsavir; INSTI, integrase inhibitor; LEN, Lenacapavir; LPV, Lopinavir; NNRTI, non‐nucleoside reverse transcriptase inhibitor; NRTI, nucleoside reverse transcriptase inhibitor; NVP, Nevirapine; PI, protease inhibitor; RAL, Raltegravir; RPV, Rilpivirine; r, Ritonavir; 3TC, Lamivudine; TDF, Tenofovir disoproxil.

**FIGURE 5:**
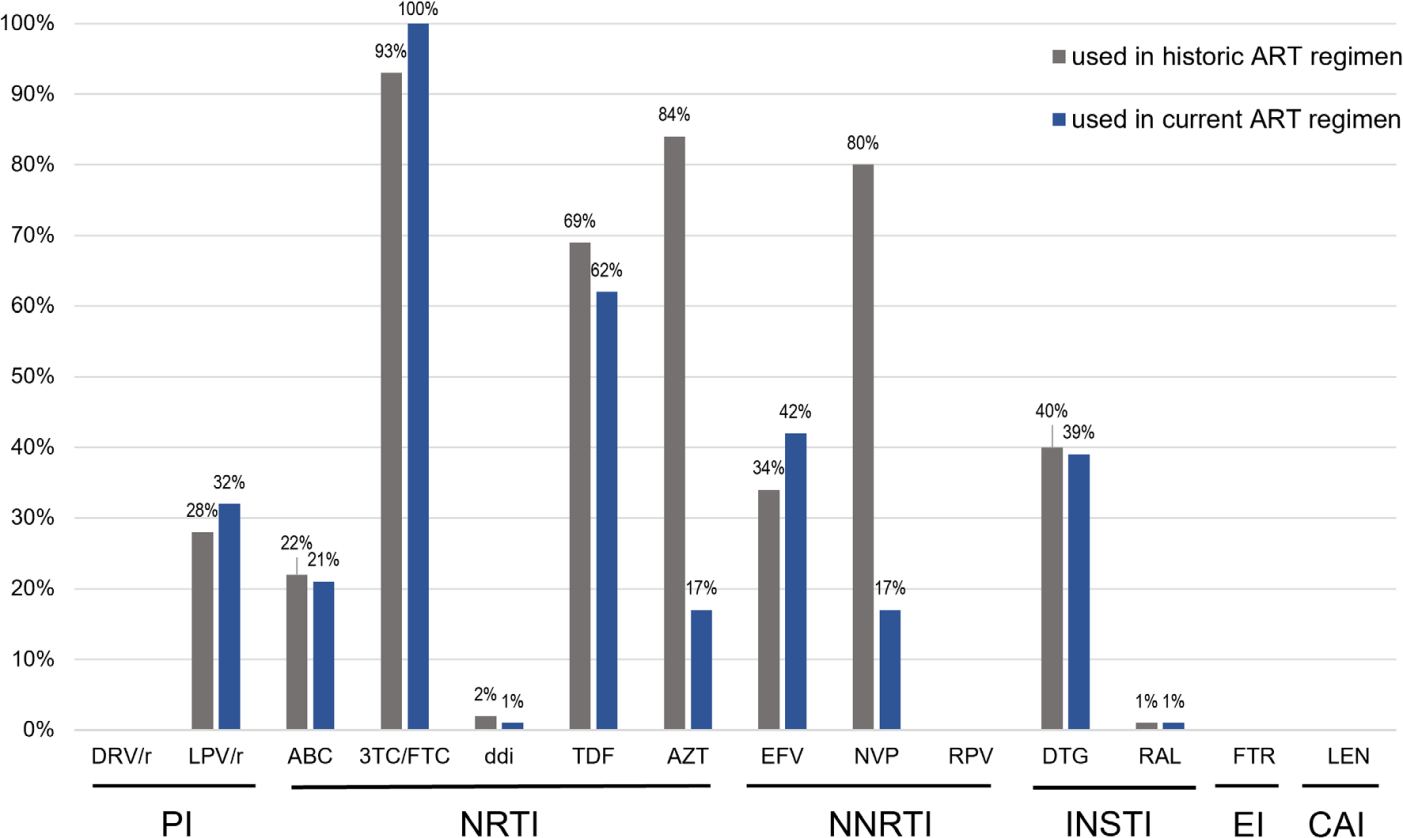
Use of ARVs of different substance classes in previous and current ART regimens within the total cohort (*n* = 510). ABC, Abacavir; ART, antiretroviral therapy; ARV, antiretroviral; AZT, Zidovudine; CAI, capsid inhibitor; ddi, Didanosin; DRV, Darunavir; DTG, Dolutegravir; EFV, Efavirenz; EI, entry inhibitor; FTC, Emtricitabine; FTR, Fostemsavir; INSTI, integrase inhibitor; LEN, Lenacapavir; LPV, Lopinavir; NNRTI, non‐nucleoside reverse transcriptase inhibitor; NRTI, nucleoside reverse transcriptase inhibitor; NVP, Nevirapine; PI, protease inhibitor; RAL, Raltegravir; RPV, Rilpivirine; r, Ritonavir; 3TC, Lamivudine; TDF, Tenofovir disoproxil.

Based on the resistance levels derived from these samples, high resistance rates, particularly for the NRTIs 3TC and FTC (56% each) and the NNRTIs EFV (62%) and NVP (72%) were shown (Figure 4). This corresponds to the ARVs most frequently used both in the past and currently (Figure 5). In the cohort, previous use of LPV/r was significantly associated with major RAMs against PIs (*p* = 0.018) (Supplement S7). EFV exposure was also significantly associated with NNRTI resistance mutations (*p* = 0.019), whereas NVP exposure was not significantly associated with NNRTI resistance mutations (*p* = 0.789). (Supplement S7). Of the 76 participants with mutations against NNRTIs, 26 had EFV, and 11 had NVP in their current ART.

However, the viruses examined were also resistant to FTR in 31% of the successfully analysed cases (Figure 4). In contrast, high susceptibility rates were detected for the INSTIs DTG (100%) and raltegravir (RAL) (100%), as well as CAIs (lenacapavir, LEN 98%) and PIs. Among the latter, darunavir (DRV) proved to be more robust than LPV, which is frequently used in ROC (100% vs. 94% susceptibility) (Figures 4 and 5).

Finally, a history of ART interruption was significantly associated with the occurrence of mutations against NRTIs (*p* = 0.036). This was not the case for any other drug class.

### Analysis of relatives

The cohort is also characterized by the fact that many of the subjects were related. Almost 40% (202/510, 39.6%) had another relative in the cohort, and 93 family connections could be examined. A total of 83 children (83/202, 41.1%), including 33 siblings (33/202, 16.3%), were included. Due to the frequent occurrence of mother–child pairs, the subgroup differed from the other participants without relatives in sex by having more women (*p* = 0.028). Mode of HIV transmission differed significantly between participants with and without relatives in the cohort (*p* = 0.046). Sexual transmission was more commonly reported among participants with relatives, while vertical transmission was slightly more frequent among those without relatives (Supplement S8).

The median VL at study inclusion among participants with relatives was 0 cop/ml (IQR 0–287), and the median CD4 count was 533/μl (IQR 267–910). However, no significant deviation from participants without relatives was apparent (VL *p* = 0.250 and CD4 *p* = 0.897) (Supplement S8). In 62 (30.7%) out of these 202 individuals, HIV viral RNA could be detected (Supplement 8). The main ART regimens used here were TDF + 3TC + DTG (67/202, 33.2%), followed by TDF + FTC + EFV (48/202, 23.8%) and ABC + 3TC + LPV/r (38/202, 18.8%) (Supplement S8). INSTI‐based regimens were used significantly less frequently among participants with relatives (*p* = 0.020), but NNRTI‐based regimens were used more frequently *(p* = 0.048) (Supplement S8). Interruptions in ART were seen significantly less frequently (*p* = 0.015), while obtaining psycho‐social care showed no significant difference (*p* = 0.768) (Supplement S8).

RAMs were not frequently observed. No major RAMs were found for INSTIs and CAIs. Among the PIs, three participants with relatives (3/202, 1.5%) showed major resistance mutations. More RAMs were detected for NRTIs and NNRTIs: with 23 detections (23/36, 63.9%), the M184V mutation was by far the most common (Supplement S6). With regard to NNRTIs, the mutation K103N (12/36, 33.3%) was the most common (Supplement S6). RAMs to FTR were also detected in eight cases, four of which were M434I (4/8, 50.0%) (Supplement S6). Analysis within families revealed concordances in only four families out of 41 families with members with detectable VL (4/41, 9.8%). This concerned two pairs of siblings, none of whose parents were included in the cohort.

### Locoregional analysis

Finally, the regional distribution of participants was analysed. The absolute majority of subjects resided in the 3rd arrondissement (128/510), followed by the 4th arrondissement (113/510) (Figure 2b and Supplement S9). These two arrondissements also had the highest proportion of participants with detectable VL (53/128, 41.4% and 38/113, 33.6%) (Figure 2c and Supplement S9). In contrast, the proportion of individuals on an INSTI‐based ART regimen was relatively low here (50/128, 39.7% and 40/113, 35.4%), whereas in the 1st and 2nd arrondissements, more than half of the participants were on such a regimen (25/49, 51.0% and 13/25, 52.0%) (Figure 2d and Supplement S9). Although these two districts accounted for the majority of participating medical facilities, they contributed the lowest number of subjects to the overall cohort (49/510 and 25/510) (Figure 2a,b and Supplement S9). The 5th arrondissement had the highest proportion (65/89, 73.0%) of participants requiring psycho‐social support (Figure 2e and Supplement S9). Interestingly, this district is relatively far from the AVENIR POSITIF base in the 1st arrondissement (Figure 2a). Drug resistance was distributed relatively evenly across the city; the 4th arrondissement had the highest proportion of participants with resistance to at least one ARV class (Figure 2f and Supplement S10). However, no significance for the differences between the districts could be determined for any of the characteristics considered.

## DISCUSSION

Our study provides one of the most comprehensive analyses of HIV‐1 drug resistance among young people living with HIV in the ROC. By combining virological, genotypic and sociobehavioral data, we observed that one‐third of participants exhibited a detectable VL, with a predominance of RAMs in (N)NRTIs. INSTI, PI and CAI resistance were rare, while the INSTI DTG remained highly effective. However, of the viremic participants, more than half showed resistance to at least one ARV. These findings highlight the evolution of HIV‐1 resistance under long‐term ART pressure in a young African population and provide insight into future treatment and prevention strategies.

The global 2024 WHO report estimated that 3.9% to 8.6% of individuals with virologic failure on DTG‐based ART exhibit INSTI resistance, emphasizing the need for continued surveillance. The same report reiterated that high NNRTI resistance, such as that seen here, remains a barrier in many low‐resource settings [1].

M184V was the most common RAM for NRTIs (54.1%), while K103N predominated among NNRTIs (40.8%). This pattern reflects historical exposure to lamivudine/emtricitabine and efavirenz/nevirapine first‐line regimens, consistent with observations in ROC and neighbouring countries [13, 14, 21, 22]. The predominant subtype A aligns with regional molecular epidemiology [6, 12, 14, 22]. We observed no major INSTI resistance mutations, reflecting the recent introduction of DTG as first‐line therapy in 2019 [23, 24]. Although resistance to PIs was rare, the risk of ART with only one effective ARV and resulting treatment failure is imminent [22]. Approximately one‐quarter of the participants in this study with detectable VL were on DTG‐containing ART. Considering the young age of the PLWH in this study (median age 19.5 years), alternative ARV classes will be essential for long‐term treatment success [25].

Noncanonical mutations were identified through NGS, and polymorphisms associated with potential resistance to new long‐acting drugs, including LEN and fostemsavir(FTR), were observed. We identified potential resistance to FTR in 31% of samples. A similar, albeit lower, mutation load (13.3%) has been described for HIV‐1 subtype C in Botswana in both ART‐naive and ART‐experienced PLWH [26]. These findings likely reflect natural viral variability rather than selective drug pressure, as neither agent is available in the region. Nevertheless, the efficacy of new ARV classes and substances appears to be important given the risk of insufficient ART with increasing multi‐class resistance and repeated virological failure [27, 28, 29, 30]. Despite robust ART using newer ARVs, delayed genotypic resistance testing may overlook existing resistance, particularly in treatment‐experienced individuals, and lead to repeated virological failure [31, 32]. This calls for routine resistance monitoring, better adherence support and preparedness for rollout of new drug classes.

The cohort is characterized by a high proportion (40%) of related participants. Among 62 individuals who had detectable VL, matching resistance patterns were seen in only four family constellations. In particular, the two pairs of siblings suggest a common viral origin, most likely through vertical transmission. The relatively low proportion of common resistance patterns despite frequently reported mother‐to‐child infection is consistent with observations and calls for an individualized therapeutic approach [33, 34]. Unexpectedly, vertical transmission appeared less common among related participants than in the overall cohort, possibly reflecting sexually acquired infections during adolescence. This developmental stage is associated with higher‐risk behaviours, while approximately 25% of participants voluntarily requesting HIV testing reflects awareness in this young population [35, 36, 37].

Behavioural factors, including ART interruption and stigma, strongly contribute to virological failure, especially in children and adolescents [25, 38, 39, 40]. This cohort also had a high rate of treatment interruptions (59%), which has hardly changed in recent years for Pointe‐Noire [41]. Psychosocial support strengthens adherence among young PLWH [42]. Institutions such as AVENIR POSITIF help to improve the treatment outcomes of participants with psychoeducational programmes. However, inconsistencies in care provision and material supply in low‐income countries must not be overlooked, alongside poor behavioural adherence, especially given the current increases in funding cuts for prevention and treatment programmes [43]. In particular, a dense care structure is increasingly being created in urban areas. For example, Pointe‐Noire has the most treatment and testing sites in ROC [44]. Interestingly, geographic disparities within Pointe‐Noire revealed that participants from central districts with higher care infrastructure density were more likely to receive INSTI‐based regimens and achieve viral suppression, while peripheral areas showed increased psychosocial support utilization but higher rates of inadequate treatment.

Our study has several limitations. This cross‐sectional analysis captured a single time point, with incomplete laboratory data—particularly CD4 counts—and limited reconstruction of treatment histories. Questionnaire‐based data are susceptible to recall bias and may lead to misclassification, particularly regarding sensitive information such as mode of HIV transmission. Sample transport and resource constraints may have affected sample quality, leading to exclusions. Although this is not expected to result in any relevant loss of information regarding the actual VL, it does highlight the general problem of insufficient supply of sub‐Saharan Africa with the required essential consumables. Furthermore, we used a modern cost‐intensive NGS system for resistance analysis that might not be used in countries with limited resources, representing also virus populations of lesser clinical relevance by applying a lower threshold than typical Sanger sequencing. Finally, there may be a selection bias, as mainly people associated with AVENIR POSITIF were included.

## CONCLUSION

The resistance patterns observed in the ROC mirror those across sub‐Saharan Africa, emphasizing the need for context‐specific strategies to sustain ART effectiveness. Strengthening adherence interventions, implementing routine resistance testing and ensuring equitable access to newer drug classes are essential. These efforts are critical to achieving global HIV targets, including the 95–95–95 goals and ending AIDS as a public health threat by 2030.

## FUNDING INFORMATION

The work in the Republic of Congo was funded by grants from European and Developing Countries Clinical Trials Partnership CANTAM (EDCTP‐CSA2020NoE‐3100) and the Alexander von Humboldt‐Stiftung. The funders had no role in the design, analysis or decision to submit this manuscript. The authors received no financial support for the authorship and/or publication of this article.

## CONFLICT OF INTEREST STATEMENT

All authors declare no conflict of interest.

## ETHICS STATEMENT

The study was planned in accordance with the World Medical Association Declaration of Helsinki and approved by the FCRM Ethics Committee under number n°034/CIE/FCRM/2021. A signed written informed consent form was obtained from each participant included in the study or their legal guardian (parent). Children aged 6–18 also signed an assent.

## Supporting information


**Supplement S1:** Demographic and clinical characteristics of the participants below 35 years of age.
**Supplement S2:** Demographic and clinical characteristics of additional participants (relatives/partners) above 35 years of age.
**Supplement S3:** Distribution of current ART regimen between PLWH with and without viral suppression.
**Supplement S4:** Distribution of INSTI‐, PI‐ and NNRTI‐based ART regimens as well as ARVs as part of current regimens between participants with and without detectable VL. The number of participants is shown.
**Supplement S5:** Demographic and clinical characteristics of participants with detectable VL and resistance analysis.
**Supplement S6:** Number of successful amplified HIV genome regions and detected resistance‐associated mutations per antiretroviral substance class in participants with detectable viral load and drug resistance analysis. The subgroup of participants with relatives in the cohort is separately presented. The most frequently detected resistance mutations associated with (N)NRTI resistance (M184V and K103N) are in bold.
**Supplement S7:** Distribution of historic use of Nevirapine, Efavirenz and Lopinavir/Ritonavir and detected (major) resistance‐associated mutations against NNRTIs and PIs, respectively. Shown are the numbers of participants.
**Supplement S8:** Demographic and clinical characteristics of participants with and without relatives in the cohort.
**Supplement S9:** Regional distribution of participants and their proportions with detectable VL, INSTI‐based ART regimen and psycho‐social support (*n* = 510, 6 missings and 2 participants living outside the city of Pointe‐Noire).
**Supplement S10:** Regional distribution of participants with resistance to at least one ARV‐class, PIs, NRTIs and NNRTIs as a proportion of the participants with detectable VL and successfully amplified HIV genome region living in the arrondissement.

## Data Availability

The data that support the findings of this study are available from the corresponding author upon reasonable request.
