# Supplementary material for: HIV drug resistance and treatment success in the Republic of Congo: Implications for optimized treatment
Source: HIV Med. 2026 May 29;27(9):1421–33. doi: 10.1111/hiv.70261 (PMC13547435; doi:10.1111/hiv.70261)
Supplement: Supplementary file 1 [file HIV-27-1421-s001.docx]

**Supplements**

| Characteristics | participating PLWH on ART ≤ 35 years | | | |
| --- | --- | --- | --- | --- |
|  | detectable  viral load  n=133 | no detected  viral load  n=223 | *p*-value | total  n=356 |
| Sex |  |  |  |  |
| Male | 53 (39.8) | 105 (47.1) | 0.184 | 158 (44.4) |
| Female | 80 (60.2) | 118 (52.9) |  | 198 (55.6) |
| Median age,  years [IQR] | 16.0 [11.5-20] | 15.0 [11-20] | 0.973 | 15.5 [11-20] |
| Median Body Mass Index,  kg/m^2^ [IQR] | 17.0 [14.3-20.4] | 18.0 [15.3-22] | **0.036** | 18.0 [15-21] |
| Employment status  employed  unemployed/other^‡^  missing | 20 (15.0)  81 (81.2)  5 (3.8) | 38 (17.0)  179 (80.3)  6 (2.7) | 0.623 | 58 (16.3)  287 (80.6)  11 (3.1) |
| Educational level |  |  |  |  |
| none/primary school | 52 (39.1) | 84 (37.7) | 0.789 | 136 (38.2) |
| secondary school/higher | 81 (60.9) | 139 (62.3) |  | 220 (61.8) |
| Marital state |  |  |  |  |
| no partner/widow | 125 (94.0) | 202 (90.6) | 0.257 | 327 (91.9) |
| in partnership | 8 (6.0) | 21 (9.4) |  | 29 (8.1) |
| Median duration of HIV infection,  years [IQR] | 9.5 [5.4-13.4] | 9.4 [4.2-12.8] | 0.375 | 9.4 [4.9-13.4] |
| Median time since ART start,  years [IQR] | 8.5 [4.7-12.6] | 9.1 [3.2-12.4] | 0.482 | 8.8 [3.8-12.5] |
| HIV type  HIV-1  HIV-2  missing | 75 (56.4)  2 (1.5)  56 (42.1) | 125 (56.1)  1 (0.4)  97 (43.5) | 0.303 | 200 (56.2)  3 (0.8)  153 (43.0) |
| Viral load at recruitment |  |  |  |  |
| Median, cop/ml [IQR] | 2,204 [366-15,190] | - | - | - |
| 40-200 cop/ml | 18 (13.5) | - | - | - |
| 201-1,000 cop/ml | 34 (25.6) | - | - | - |
| > 1,000 cop/ml | 81 (60.9) | - | - | - |
| Median last CD4 cell count,  /µl [IQR] (missing n=231) | 479 [302-886] | 659 [332-1,000] | 0.330 | 572 [311-917] |
| < 12 months before recruitment (n=4) | 4,890 (n=1) | 212 [117-212] |  | 607 [141-3,918] |
| ≥ 12 months before recruitment (n=121) | 478 [302-870] | 659 [353-1,000] |  | 569 [317-910] |
| WHO HIV stage |  |  |  |  |
| stage 1  stage 2  stage 3 | 108 (81.2)  13 (9.8)  12 (9.0) | 199 (89.2)  13 (5.8)  11 (4.9) | 0.103 | 307 (86.2)  26 (7.3)  23 (6.5) |
| HIV mode of transmission  vertical  sexual  other/unknown | 94 (70.7)  8 (6.0)  31 (23.3) | 161 (72.2)  24 (10.8)  38 (17.0) | 0.187 | 255 (71.6)  32 (9.0)  69 (19.4) |
| Reason for initial HIV test  self-initiated/voluntary  other  unknown | 26 (19.5)  48 (36.1)  59 (44.4) | 58 (26.0)  81 (36.3)  84 (37.7) | 0.350 | 84 (23.6)  129 (36.2)  143 (40.2) |
| HIV state mother  negative  positive  unknown | 8 (6.0)  83 (62.4)  42 (31.6) | 24 (10.8)  142 (63.7)  57 (25.6) | 0.189 | 32 (9.0)  225 (63.2)  99 (27.8) |
| HIV state father  negative  positive  unknown | 32 (24.1)  22 (16.5)  79 (59.4) | 65 (29.1)  52 (23.3)  106 (47.5) | 0.651 | 97 (27.2)  74 (20.8)  185 (52.0) |
| HIV/TB co-infection | 23 (17.3) | 33 (14.8) | 0.566 | 56 (15.7) |
| HIV/HBV co infection | 1 (0.8) | - | - | 1 (0.3) |
| HIV/HCV co-infection | - | - | - | - |
| Interruption of ART in the past | 89 (66.9) | 109 (48.9) | **0.027** | 198 (55.6) |
| Median number of ART regimens  [IQR] | 2 [2-2] | 2 [2-2] | 0.399 | 2 [2-2] |
| Median number of tablets daily taken, [IQR] | 2 [1-4] | 1 [1-4] | **0.021** | 1 [1-4] |
| Patient of AVENIR POSITIF  yes  no  missing | 112 (84.2)  2 (1.5)  19 (14.3) | 164 (73.5)  -  59 (26.5) | 0.089  **(0.020^†^)** | 276 (77.5)  2 (0.6)  78 (21.9) |
| Obtaining psycho-social support  yes | 112 (84.2) | 164 (73.5) | 0.269 | 276 (77.5) |
| therapy education | 110 (82.7) | 156 (70.0) | 0.178 | 266 (74.7) |

**Supplement 1:** Demographic and clinical characteristics of the participants under 35 years of age.

Categorical variables, n (%), were compared by χ²-test for independence. Comparison of medians used Mann-Whitney-U-test. Statistical significance considered at *p*-values <0.05, demanding 95% confidence intervals.

^‡^ including students, housewives and retired

^†^ *p*-value, if missing cases were classified as “no”

PLWH, people living with HIV; ART, antiretroviral therapy; IQR, interquartile range; ENV, HIV envelope gene; WHO, World Health Organization; TB, tuberculosis; HBV, hepatitis B virus; HCV, hepatitis C virus.

| Characteristics | participating PLWH on ART (relatives/partners > 35 years) | | | |
| --- | --- | --- | --- | --- |
|  | detectable  viral load  n=40 | no detected  viral load  n=114 | *p*-value | total  n=154 |
| Sex |  |  |  |  |
| Male | 12 (30.0) | 29 (25.4) | 0.576 | 41 (26.6) |
| Female | 28 (70.0) | 85 (74.6) |  | 113 (73.4) |
| Median age,  years [IQR] | 44.5 [41-52.8] | 46.0 [42-52] | 0.546 | 46.0 [42-52] |
| Median Body Mass Index,  kg/m^2^ [IQR] | 22.5 [20-27.8] | 24.0 [20-28] | 0.965 | 23.7 [20-28] |
| Employment status  employed  unemployed/other^‡^ | 25 (62.5)  15 (37.5) | 70 (61.4)  44 (38.6) | 0.903 | 95 (61.7)  59 (38.3) |
| Educational level |  |  |  |  |
| none/primary school | 6 (15.0) | 21 (18.4) | 0.626 | 27 (17.5) |
| secondary school/higher | 34 (85.0) | 93 (84.2) |  | 127 (82.5) |
| Marital state |  |  |  |  |
| no partner/widow | 18 (45.0) | 57 (50.0) | 0.587 | 75 (48.7) |
| in partnership | 22 (55.0) | 57 (50.0) |  | 79 (51.3) |
| Median duration of HIV infection,  years [IQR] | 10.0 [4.5-13.5] | 11.5 [6.3-15.4] | 0.184 | 11.4 [5.5-14.5] |
| Median time since ART start,  years [IQR] | 9.5 [4.5-13.3] | 11.1 [5.5-14.2] | 0.167 | 10.5 [5.4-13.5] |
| HIV type  HIV-1  HIV-2  missing | 22 (55.0)  -  18 (45.0) | 65 (57.0)  -  49 (43.0) | 0.884  - | 87 (56.5)  -  67 (43.5) |
| Viral load at recruitment |  |  |  |  |
| Median, cop/ml [IQR] | 4,488 [743-24,229] | - | - | - |
| 40-200 cop/ml | 5 (12.5) | - | - | - |
| 201-1,000 cop/ml | 7 (17.5) | - | - | - |
| > 1,000 cop/ml | 28 (70.0) | - | - | - |
| Median last CD4 cell count,  /µl [IQR] (missing n=138) | 80 [5-80] | 436 [166-561] | 0.611 | 355 [89-591] |
| < 12 months before recruitment (n=3) | 703 [5-703] | 13 (n=1) |  | 13 [5-703]* |
| ≥ 12 months before recruitment (n=13) | 80 (n=1) | 460 [219-591] |  | 436 [166-561] |
| WHO HIV stage |  |  |  |  |
| stage 1  stage 2  stage 3 | 36 (90.0)  2 (5.0)  2 (5.0) | 105 (92.1)  5 (4.4)  4 (3.5) | 0.901 | 141 (91.6)  7 (4.5)  6 (3.9) |
| HIV mode of transmission  vertical  sexual  other/unknown | 1 (2.5)  38 (95.0)  1 (2.5) | 1 (0.9)  108 (94.7)  5 (4.4) | 0.446 | 2 (1.3)  146 (94.8)  6 (3.9) |
| Reason for initial HIV test  self-initiated/voluntary  other  unknown | 10 (25.0)  14 (35.0)  16 (40.0) | 27 (23.7)  36 (31.6)  51 (44.7) | 0.921 | 37 (24.0)  50 (32.5)  67 (43.5) |
| HIV state mother  negative  positive  unknown | 12 (30.0)  2 (5.0)  26 (65.0) | 44 (38.6)  3 (2.6)  67 (58.8) | 0.348 | 56 (36.4)  5 (3.2)  93 (60.4) |
| HIV state father  negative  positive  unknown | 8 (20.0)  -  32 (80.0) | 39 (34.2)  1 (0.9)  74 (64.9) | 0.655 | 47 (30.5)  1 (0.6)  106 (68.8) |
| HIV/TB co-infection | 8 (20.00) | 33 (28.9) | 0.345 | 41 (26.6) |
| HIV/HBV co infection | - | - | - | - |
| HIV/HCV co-infection | - | 1 (0.9) | - | 1 (0.6) |
| Interruption of ART in the past | 26 (65.0) | 77 (67.5) | 0.866 | 103 (66.9) |
| Median number of ART regimens  [IQR] | 2 [2-2] | 2 [2-2] | 0.904 | 2 [2-2] |
| Median number of tablets daily taken, [IQR] | 1 [1-2] | 1 [1-2] | 0.784 | 1 [1-2] |
| Patient of AVENIR POSITIF  yes  no  missing | 15 (37.5)  -  25 (62.5) | 47 (41.2)  1 (0.9)  66 (57.9) | 0.576  (0.680^†^) | 62 (40.3)  1 (0.6)  91 (59.1) |
| Obtaining psycho-social support  yes | 15 (37.5) | 48 (42.1) | 0.695 | 63 (40.9) |
| therapy education | 13 (32.5) | 44 (38.6) | 0.586 | 57 (37.0) |

**Supplement 2:** Demographic and clinical characteristics of additional participants (relatives/partners) above 35 years of age.

Categorical variables, n (%), were compared by χ²-test for independence. Comparison of medians used Mann-Whitney-U-test. Statistical significance considered at *p*-values <0.05, demanding 95% confidence intervals.

* range

^‡^ including students, housewives and retired

^†^ *p*-value, if missing cases were classified as “no”

PLWH, people living with HIV; ART, antiretroviral therapy; IQR, interquartile range; ENV, HIV envelope gene; WHO, World Health Organization; TB, tuberculosis; HBV, hepatitis B virus; HCV, hepatitis C virus.

| Current ART regimen | participating PLWH on ART | | |
| --- | --- | --- | --- |
|  | detectable  viral load  n=173 | no detected  viral load  n=337 | total  n=510 |
| ABC+3TC+DTG | - | 2 (0.6) | 2 (0.4) |
| ABC+3TC+EFV | 3 (1.7) | 7 (2.1) | 10 (2.0) |
| ABC+3TC+LPV/r | 31 (17.9) | 48 (14.2) | 79 (15.5) |
| ABC+3TC+NVP | 2 (1.2) | 1 (0.3) | 3 (0.6) |
| ABC+ddi+LPV/r | - | 1 (0.3) | 1 (0.2) |
| AZT+3TC | - | 1 (0.3) | 1 (0.2) |
| AZT+3TC+DTG | - | 2 (0.6) | 2 (0.4) |
| AZT+3TC+EFV | 5 (2.9) | 8 (2.4) | 13 (2.5) |
| AZT+3TC+LPV/r | 5 (2.9) | 6 (1.8) | 11 (2.2) |
| AZT+3TC+NVP | 21 (12.1) | 26 (7.7) | 47 (9.2) |
| DRV/r+RAL | - | 1 (0.3) | 1 (0.2) |
| TDF+3TC+DTG | 46 (26.6) | 151 (44.8) | 197 (38.6) |
| TDF+3TC+EFV | - | 3 (0.9) | 3 (0.6) |
| TDF+FTC+EFV | 39 (22.5) | 57 (16.9) | 96 (18.8) |
| TDF+FTC+LPV/r | 21 (12.1) | 21 (6.2) | 42 (8.2) |
| missing | - | 2 (0.6) | 2 (0.4) |

**Supplement 3:** Distribution of current ART regimen between PLWH with and without viral suppression.

n (%)

PLWH, people living with HIV; ART, antiretroviral therapy; ABC, Abacavir; 3TC, Lamivudine; DTG, Dolutegravir; EFV, Efavirenz; LPV, Lopinavir; r, Ritonavir; NVP, Nevirapine; ddi, Didanosin; AZT, Zidovudine; DRV, Darunavir; RAL, Raltegravir; TDF, Tenofovir disoproxil; FTC, Emtricitabine.

| **ART** | **viral load detected** | | χ^2^  (p-value) |
| --- | --- | --- | --- |
|  | yes | no |  |
| INSTI-based  yes  no | 46  127 | 156  179 | 19.009  **(<0.001)** |
|  |  |  |  |
| PI-based  yes  no | 57  116 | 77  258 | 5.831  **(0.016)** |
|  |  |  |  |
| NNRTI-based  yes  no | 70  103 | 102  233 | 5.109  (0.024) |
|  |  |  |  |
|  |  |  |  |
| **ARV as part of ART regimen** |  |  |  |
| Zidovudine  yes  no | 31  142 | 43  294 | 2.453  (0.177) |
| Didanosine  yes  no | 0  173 | 1  336 | 0.514  (0.473) |
| Abacavir  yes  no | 36  137 | 59  278 | 0.822  (0.365) |
| Lamivudine  yes  no | 113  60 | 255  82 | 6.095  **(0.014)** |
| Tenofovir  yes  no | 106  67 | 232  105 | 2.932  (0.087) |
| Emtricitabine  yes  no | 60  113 | 78  259 | 7.709  (0.005) |
| Nevirapine  yes  no | 23  159 | 27  310 | 3.608  (0.058) |
| Efavirenz  yes  no | 47  126 | 75  262 | 1.516  (0.218) |
| Lopinavir/Ritonavir  yes  no | 57  116 | 76  261 | 6.409  **(0.011)** |
| Dolutegravir  yes  no | 46  127 | 155  182 | 18.026  **(<0.001)** |
| Raltegravir  yes  no | 0  173 | 1  336 | 0.514  (0.473) |

**Supplement 4:** Distribution of INSTI-, PI- and NNRTI-based ART regimens as well as ARVs as part of current regimens between participants with and without detectable VL. Shown are the numbers of participants.

Comparison was performed by χ²-test for independence. P-values <0.05 were considered statistically significant, demanding 95% confidence intervals.

ART, antiretroviral therapy; INSTI, integrase inhibitor; PI, protease inhibitor; NNRTI, non-nucleoside reverse transcriptase inhibitor; ARV, antiretroviral

| Characteristics | participating PLWH on ART  with detectable VL and resistance analysis | | | |
| --- | --- | --- | --- | --- |
|  | no HIVDR  n=62 | HIVDR  n=85 | *p*-value | total  n=147 |
| Sex |  |  |  |  |
| Male | 22 (35.5) | 27 (31.8) | 0.638 | 49 (33.3) |
| Female | 40 (64.5) | 58 (68.2) |  | 98 (66.7) |
| Median age,  years [IQR] | 18.0 [12.8-28.3] | 18.0 [14-40] | 0.367 | 18.0 [13-34] |
| Median Body Mass Index,  kg/m^2^ [IQR] | 19.0 [15-22] | 18.4 [16-22] | 0.581 | 18.9 [15-22] |
| Employment status  employed  unemployed/other^‡^  missing | 15 (24.2)  47 (75.8)  - | 23 (27.1)  60 (70.6)  2 (2.4) | 0.635 | 38 (25.8)  107 (72.8)  2 (1.4) |
| Educational level |  |  |  |  |
| none/primary school | 18 (29.0) | 30 (35.3) | 0.427 | 48 (32.7) |
| secondary school/higher | 44 (71.0) | 55 (64.7) |  | 99 (67.3) |
| Marital state |  |  |  |  |
| no partner/widow | 52 (83.9) | 69 (81.2) | 0.674 | 121 (82.3) |
| in partnership | 10 (16.1) | 16 (18.8) |  | 26 (17.7) |
| Median duration of HIV infection,  years [IQR] | 10.0 [5.4-13.4] | 9.9 [5.6-13.4] | 0.772 | 9.9 [5.4-13.4] |
| Median time since ART start,  years [IQR] | 8.5 [4.8-11.5] | 9.4 [5.0-13.4] | 0.221 | 8.9 [4.9-12.8] |
| HIV type  HIV-1  HIV-2  missing | 36 (58.1)  -  26 (41.9) | 47 (55.3)  2 (2.4)  36 (42.4) | 0.223 | 83 (56.5)  2 (1.4)  62 (42.2) |
| HIV tropism  R5  X4  missing | 10 (16.1)  7 (11.3)  45 (72.6) | 45 (52.9)  10 (11.8)  30 (35.3) | 0.053 | 55 (37.4)  17 (11.6)  75 (51.0) |
| HIV ENV subtype |  |  |  |  |
| A1 | 9 (14.5 | 22 (25.9) | 0.470 | 31 (21.1) |
| A2 | - | 1 (1.2) |  | 1 (0.7) |
| A3 | 3 (4.8) | 7 (8.2) |  | 10 (6.8) |
| A7 | - | 1 (1.2) |  | 1 (0.7) |
| C | 1 (1.6) | - |  | 1 (0.7) |
| D | 2 (3.2) | 3 (3.5) |  | 5 (3.4) |
| F1 | - | 3 (3.5) |  | 3 (2.0) |
| G | 5 (8.1) | 12 (14.1) |  | 17 (11.6) |
| H | 2 (3.2) | 16 (18.8) |  | 18 (12.2) |
| missing | 40 (64.5) | - |  | 60 (40.8) |
| Viral load at recruitment |  |  |  |  |
| Median, cop/ml [IQR] | 588  [219-6,684] | 6,164  [1,886-26,522] | **<0.001** | 3,334  [402-18,362] |
| 40-200 cop/ml | 15 (24.2) | 4 (4.7) |  | 19 (12.9) |
| 201-1,000 cop/ml | 25 (40.3) | 8 (9.4) |  | 33 (22.4) |
| > 1,000 cop/ml | 22 (35.5) | 73 (85.9) |  | 95 (64.6) |
| Median last CD4 cell count,  /µl [IQR] (missing n=100) | 500 [362-924] | 400 [256-730] | 0.065 | 462 [277-826] |
| < 12 months before recruitment (n=3) | 3,145  [1,400-3,145] | 5 (n=1) |  | 1,400  [5-1,400] |
| ≥ 12 months before recruitment (n=44) | 481 [343-846] | 427 [263-761] |  | 460 [283-788] |
| WHO HIV stage |  |  |  |  |
| stage 1  stage 2  stage 3 | 51 (82.3)  6 (9.7)  5 (8.1) | 72 (84.7)  8 (9.4)  5 (5.9) | 0.870 | 123 (83.7)  14 (9.5)  10 (6.8) |
| HIV mode of transmission  vertical  sexual  other/unknown | 35 (56.5)  14 (22.6)  13 (21.0) | 45 (52.9)  26 (30.6)  14 (16.5) | 0.360 | 80 (54.4)  40 (27.2)  27 (18.4) |
| Reason for initial HIV test  self-initiated/voluntary  other  unknown | 13 (21.0)  25 (40.3)  24 (38.7) | 20 (23.5)  26 (30.6)  39 (45.9) | 0.390 | 33 (22.4)  51 (34.7)  63 (42.9) |
| HIV state mother  negative  positive  unknown | 8 (12.9)  32 (51.9)  22 (35.5) | 11 (12.9)  38 (44.7)  36 (42.4) | 0.780 | 19 (12.9)  70 (47.6)  58 (39.5) |
| HIV state father  negative  positive  unknown | 10 (16.1)  11 (17.7)  41 (66.1) | 20 (23.5)  10 (11.8)  55 (64.7) | 0.178 | 30 (20.4)  21 (14.3)  96 (65.3) |
| HIV/TB co-infection | 8 (12.9) | 17 (20.0) | 0.303 | 25 (17.0) |
| HIV/HBV co infection | 1 (1.6) | - | 0.242 | 1 (0.7) |
| HIV/HCV co-infection | - | - |  | - |
| Interruption of ART in the past | 41 (66.1) | 57 (67.1) | 0.946 | 98 (66.7) |
| Median number of ART regimens  [IQR] | 2 [2-2] | 2 [2-2] | 0.375 | 2 [2-2] |
| Median number of tablets daily taken, [IQR] | 2 [1-4] | 1 [1-3] | 0.127 | 1 [1-4] |
| Patient of AVENIR POSITIF  yes  no  missing | 49 (79.0)  -  13 (21.0) | 58 (68.2)  1 (1.2)  26 (30.6) | 0.362  (0148^†^) | 107 (72.8)  1 (0.7)  39 (26.5) |
| Obtaining psycho-social support  yes | 49 (79.0) | 58 (68.2) | 0.449 | 107 (72.8) |
| therapy education | 48 (77.4) | 56 (65.9) | 0.412 | 104 (70.7) |

**Supplement 5:** Demographic and clinical characteristics of participants with detectable VL and resistance analysis.

Categorical variables, n (%), were compared by χ²-test for independence. Comparison of medians used Mann-Whitney-U-test. Statistical significance considered at *p*-values <0.05, demanding 95% confidence intervals.

^‡^ including students, housewives and retired

^†^ *p*-value, if missing cases were classified as “no”

PLWH, people living with HIV; ART, antiretroviral therapy; IQR, interquartile range; ENV, HIV envelope gene; WHO, World Health Organization; TB, tuberculosis; HBV, hepatitis B virus; HCV, hepatitis C virus.

|  | participating PLWH on ART with detectable viral load and drug resistance analysis | |
| --- | --- | --- |
|  | **total**  n=147 | with relatives in the cohort  n=62 |
| Successful amplified PrRT region | **98 (66.7)** | 36 (58.1) |
| PIs  Major mutations  D30N  I50V  I54V  I147I  L76V  M46I  V82A  V82I  V82S  Accessory mutations | 1 (1.0)  1 (1.0)  4 (4.1)  1 (1.0)  1 (1.0)  7 (7.1)  3 (3.1)  1 (1.0)  2 (2.0)  - | 1 (2.8)  -  2 (5.6)  1 (2.8)  1 (2.8)  3 (8.3)  2 (5.6)  1 (2.8)  -  - |
| NRTIs  A62A  D67N  D67G  E44D  K65R  K70G  K70N  K70R  K70T  K219E  K219Q  K219R  L74I  L74V  L210W  M41L  M184I  M184V  S68G  T69del  T69D  T215F  T215I  T215Y  V75I  V75M  Y115F | 2 (2.0)  16 (16.3)  1(1.0)  1(1.0)  5 (5.1)  1 (1.0)  1 (1.0)  12 (12.2)  1 (1.0)  4 (4.1)  9 (9.2)  6 (6.1)  2 (2.0)  2 (2.0)  4 (4.1)  12 (12.2)  2 (2.0)  **53 (54.1)**  8 (8.2)  2 (2.0)  2 (2.0)  10 (10.2)  5 (5.1)  15 (15.3)  2 (2.0)  5 (5.1)  5 (5.1) | -  6 (16.7)  -  -  3 (8.3)  1 (2.8)  -  6 (16.7)  1 (2.8)  4 (11.1)  3 (8.3)  2 (5.6)  1 (2.8)  1 (2.8)  -  3 (8.3)  1 (2.8)  23 (63.9)  3 (8.3)  1 (2.8)  -  5 (13.9)  3 (8.3)  3 (8.3)  -  1 (2.8)  2 (5.6) |
| NNRTIs  A98G  E138A  E138G  E138Q  E138K  F227L  G190A  H221Y  K101E  K101H  K101P  K103N  K103S  K238T  L100I  L234I  P225H  V106A  V106I  V106M  V108I  V179D  V179E  V179F  V179L  V179T  Y181C  Y188H  Y188L  Y318F | 13 (13.3)  4 (4.1)  5 (5.1)  3 (3.1)  1 (1.0)  2 (2.0)  20 (20.4)  11 (11.2)  11 (11.2)  2 (2.0)  5 (5.1)  **40 (40.8)**  7 (7.1)  3 (3.1)  2 (2.0)  1 (1.0)  6 (6.1)  1 (1.0)  1 (1.0)  1 (1.0)  15 (15.3)  2 (2.0)  2 (2.0)  1 (1.0)  3 (3.1)  2 (2.0)  11 (11.2)  2 (2.0)  4 (4.1)  7 (7.1) | 6 (16.7)  -  1 (2.8)  1 (2.8)  1 (2.8)  1 (2.8)  7 (19.4)  6 (16.7)  3 (8.3)  2 (5.6)  3 (8.3)  12 (33.3)  1 (2.8)  1 (2.8)  1 (2.8)  1 (2.8)  2 (5.6)  -  -  1 (2.8)  5 (13.9)  1 (2.8)  1 (2.8)  1 (2.8)  1 (2.8)  -  3 (8.3)  3 (8.3)  3 (8.3)  4 (11.1) |
| Successful amplified INT region | **95 (64.6)** | 33 (53.2) |
| INSTIs  Major mutations  Accessory mutations  E157Q  G149A  L74M  S153A  T97A | -  1 (1.1)  1 (1.1)  2 (2.1)  1 (1.2)  3 (3.2) | -  -  -  -  -  2 (6.1) |
| Successful amplified GAG region | **98 (66.7)** | 38 (61.3) |
| CAIs  Major mutations  Accessory mutations  T107A | -  2 (2.0) | -  - |
| Successful amplified ENV region | **85 (57.8)** | 35 (56.5) |
| FTR  M426L  M434I  M475I  S375I  S375M  S375T  V506R | 2 (2.4)  15 (17.6)  1 (1.2)  4 (4.7)  4 (4.7)  2 (2.4)  1 (1.2) | 1 (2.9)  4 (11.4)  1 (2.9)  1 (2.9)  1 (2.9)  -  - |

**Supplement 6:** Number of successful amplified HIV genome regions and detected resistance associated mutations per antiretroviral substance class in participants with detectable viral load and drug resistance analysis. The subgroup of participants with relatives in the cohort is separately presented. The most frequently detected resistance mutations associated with (N)NRTI resistance (M184V and K103N) are in bold.

n (%); percentages of amplified regions refer to the number of analysed samples, while percentages of mutations refer to the number of samples with the corresponding, successfully amplified region.

PLWH, people living with HIV; ART, antiretroviral therapy; PrRT, protease reverse transcriptase ; PI, protease inhibitor; NRTI, nucleoside reverse transcriptase inhibitor; NNRTI, non-nucleoside reverse transcriptase inhibitor; INSTI, integrase; INI, integrase inhibitor; GAG, group specific antigen; CAI, capsid inhibitor; ENV, envelope; FTR, Fostemsavir.

| **ARV in the past** | **resistance associated mutations**  **against NNRTIs** | | χ^2^  (p-value) |
| --- | --- | --- | --- |
|  | yes | no |  |
| Nevirapine  yes  no | 64  12 | 18  4 | 0.071  (0.789) |
| Efavirenz  yes  no | 35  41 | 4  18 | 5.532  **(0.019)** |
|  | **major resistance associated mutations against PIs** | |  |
|  | yes | no |  |
| Lopinavir/Ritonavir  yes  no | 6  3 | 25  64 | 5.624  **(0.018)** |

**Supplement 7:** Distribution of historic use of Nevirapine, Efavirenz and Lopinavir/Ritonavir and detected (major) resistance associated mutations against NNRTIs and PIs, respectively. Shown are the numbers of participants.

Comparison was performed by χ²-test for independence. P-values <0.05 were considered statistically significant, demanding 95% confidence intervals.

ART, antiretroviral therapy; INSTI, integrase inhibitor; PI, protease inhibitor; NNRTI, non-nucleoside reverse transcriptase inhibitor; ARV, antiretroviral

| Characteristics | participating PLWH with relatives in the cohort | | | |
| --- | --- | --- | --- | --- |
|  | yes  n=202 | no  n=308 | *p*-value | |
| Sex |  |  |  |  |
| Male | 67 (33.2) | 132 (42.9) | 0.028 | |
| Female | 135 (66.8) | 176 (57.1) |  |  |
| Kinship |  |  |  |  |
| mother | 64 (31.7) | - | - | |
| father | 7 (3.5) | - | - | |
| child | 83 (41.1) | - | - | |
| sibling | 33 (16.3) | - | - | |
| partner | 20 (9.9) | - | - | |
| Viral load detected |  |  |  | |
| yes | 62 (30.7) | 111 (36.0) | 0.212 | |
| no | 140 (69.3 | 197 (64.0) |  |  |
| Median viral load at recruitment, cop/ml [IQR] | 0 [0-287] | 0 [0-788] | 0.223 | |
| Median last CD4 cell count,  /µl [IQR] | 533 [267-910] | 538 [302-900] | 0.805 | |
| HIV mode of transmission  vertical  sexual  other/unknown | 99 (49.0)  84 (41.6)  19 (9.4) | 158 (51.3)  95 (30.8)  55 (17.9) | 0.046 | |
| Interruption of ART in the past | 106 (52.2) | 195 (63.3) | 0.015 | |
| Obtaining psycho-social support | 133 (65.8) | 206 (67.1) | 0.768 | |
| ART  INSTI-based  PI-based  NNRTI-based | 67 (33.5)  55 (27.5)  78 (39.0) | 135 (43.8)  79 (25.6)  94 (30.5) | 0.020  0.644  0.048 | |
| Current ART regimen  ABC+3TC+DTG  ABC+3TC+EFV  ABC+3TC+LPV/r  ABC+3TC+NVP  ABC+ddI+LPV/r  AZT+3TC  AZT+3TC+DTG  AZT+3TC+EFV  AZT+3TC+LPV/r  AZT+3TC+NVP  DRV+r+RAL  DTG+3TC+TDF  EFV+FTC+TDF  TDF+3TC+EFV  TDF+FTC+EFV  TDF+FTC+LPV/r  missing | -  5 (2.5)  38 (18.8)  -  -  -  -  4 (2.0)  2 (1.0)  20 (9.9)  -  67 (33.2)  1 (0.5)  -  48 (23.8)  15 (7.4)  2 (1.0) | 2 (0.6)  5 (1.6)  41 (13.3)  3 (1.0)  1 (0.3)  1 (0.3)  2 (0.6)  9 (2.9)  9 (2.9)  27 (8.8)  1 (0.3)  130 (42.2)  -  3 (1.0)  47 (15.3)  27 (8.8)  - |  | |

**Supplement 8:** Demographic and clinical characteristics of participants with and without relatives in the cohort.

n (%)

Comparison was performed by χ²-test for independence. P-values <0.05 were considered statistically significant, demanding 95% confidence intervals.

PLWH, people living with HIV; ART, antiretroviral therapy; INSTI, integrase inhibitor; PI, protease inhibitor; NNRTI, non-nucleoside reverse transcriptase inhibitor; ABC, Abacavir; 3TC, Lamivudine; DTG, Dolutegravir; EFV, Efavirenz; LPV, Lopinavir; r, Ritonavir; NVP, Nevirapine; ddi, Didanosin; AZT, Zidovudine; DRV, Darunavir; RAL, Raltegravir; TDF, Tenofovir disoproxil; FTC, Emtricitabine.

| **Arrondissement** | **Participants living in the arrondissement** | **Proportion with detectable VL** | **Proportion with INSTI-based ART regimen** | **Proportion with psycho-social support** |
| --- | --- | --- | --- | --- |
| 1 (Lumumba) | 49 | 16 (32.7) | 25 (51.0) | 28 (57.1) |
| 2 (Mvou-Mvou) | 25 | 7 (28.0) | 13 (52.0) | 16 (64.0) |
| 3 (Tié-Tié) | 128 | 53 (41.4) | 50 (39.7) | 83 (64.8) |
| 4 (Loandjili) | 113 | 38 (33.6) | 40 (35.4) | 76 (67.3) |
| 5 (Mongo Mpoukou) | 89 | 24 (27.0) | 28 (31.5) | 65 (73.0) |
| 6 (Ngoyo) | 98 | 32 (32.7) | 45 (45.9) | 65 (67.0) |

**Supplement 9:** Regional distribution of participants and their proportions with detectable VL, INSTI-based ART regimen and psycho-social support (n=510, 6 missings and 2 participants living outside the city of Pointe-Noire).

n (%)

VL, viral load; INSTI, integrase inhibitor; ART, antiretroviral therapy.

| **Arrondissement** | **Proportion with resistance to at least one ARV class** | **Proportion with resistance to PIs** | **Proportion with resistance to NRTIs** | **Proportion with resistance to NNRTIs** |
| --- | --- | --- | --- | --- |
| 1 (Lumumba) | 7 (58.3) | - (0.0) | 5 (62.5) | 5 (62.5) |
| 2 (Mvou-Mvou) | 4 (57.1) | 1 (25.0) | 3 (75.0) | 4 (100.0) |
| 3 (Tié-Tié) | 26 (56.5) | 2 (6.5) | 19 (61.3) | 26 (83.9) |
| 4 (Loandjili) | 20 (66.7) | 2 (9.5) | 14 (66.7) | 17 (81.0) |
| 5 (Mongo Mpoukou) | 11 (52.4) | 1 (7.1) | 6 (42.9) | 10 (71.4) |
| 6 (Ngoyo) | 16 (55.2) | 3 (16.7) | 9 (50.0) | 14 (77.8) |

**Supplement 10:** Regional distribution of participants with resistance to at least one ARV-class, PIs, NRTIs and NNRTIs as proportion of the participants with detectable VL and successfully amplified HIV genome region living in the arrondissement.

n (%)

ARV, antiretroviral; PI, protease inhibitor; NRTI, nucleoside reverse transcriptase inhibitor; NNRTI, non-nucleoside reverse transcriptase inhibitor; VL, viral load;
